# Big progress for small subunits: new Rubisco mutants in Arabidopsis

**DOI:** 10.1093/jxb/eraa360

**Published:** 2020-10-05

**Authors:** Amanda P Cavanagh

**Affiliations:** School of Life Sciences, University of Essex, Wivenhoe Park, Colchester, UK

**Keywords:** Arabidopsis, photosynthesis, Rubisco

## Abstract

This article comments on:

**Khumsupan P, Kozlowska MA, Orr DJ, Andreou AI, Nakayama N, Patron N, Carmo-Silva E, McCormick AJ**. 2020. Generating and characterizing single- and multigene mutants of the Rubisco small subunit family in Arabidopsis. Journal of Experimental Botany **71**, 5963–5975.

**Nearly 75 years after Rubisco was first isolated from spinach leaves, the enzyme’s function and regulation remain an important research topic and longstanding target to improve plant productivity. In plants, Rubisco is a complex made up of eight large subunits (LSUs) encoded in the chloroplast genome (*rbcL*), while eight small subunits (SSUs) are encoded in the nucleus as a multi-*rbcS* gene family. Despite decades of investigation, the function and interaction of the different small subunits in plant Rubisco remain enigmatic. Khumsupan *et al.* (2020) have used CRISPR/Cas9 [clustered regularly interspaced palindromic repeats (CRISPR)/CRISPR-associated protein 9] and T-DNA insertion lines within *Arabidopsis thaliana* to generate a suite of single and multiple gene knockout mutants for the four *rbc*S members. This enabled characterization of the homogenous Rubisco pool consisting of a single SSU isoform *in planta*. In doing so, they provide a powerful toolkit to expand our understanding of Rubisco structure–function relationships inside the leaf.**

Engineering increased photosynthetic capacity has shown improvements in both model and food crops under field conditions, reigniting strategies to optimize or redesign photosynthesis to increase crop yields sustainably (Kromdijk *et al.*, 2016; Ermakova *et al.*, 2019; South *et al.*, 2019). A persistent target for improvement aims to overcome the inefficiencies of the central carbon-fixing enzyme in photosynthesis, Rubisco. Rubisco has a slow catalytic rate and also catalyses a competing oxygenation reaction, promoting the energetically intensive photorespiration cycle. Accordingly, optimizing the regulation and amount of Rubisco can result in improved crop growth and yield (Parry *et al.*, 2012; Salesse-Smith *et al.*, 2018; Yoon *et al.*, 2020). In recent years, surveys of both plant species and other photosynthetic organisms indicate that there is also exploitable variation in Rubisco performance that could be an important target for crop improvement (Sharwood *et al.*, 2016; Young *et al.*, 2016; Orr *et al.*, 2016; Galmés *et al.*, 2019), but our understanding of the structure–function relationships underpinning this variation remains fairly cryptic, and limits our ability to mine this resource.

Evidence across plant and algal Rubiscos has demonstrated that structural changes in both the LSU, where the catalytic site resides at the interface between dimers, as well as the SSU can impact the Rubisco carboxylation rate, substrate specificity, and multimer assembly (well reviewed in the accompanying article). A long-awaited goal for the Rubisco research community was realized with the expression of a recombinant Arabidopsis Rubisco in *Escherichia coli* (Aigner *et al.*, 2017). This has accelerated the ability to explore structure–function relationships across Rubisco, and has in fact been harnessed to demonstrate that the kinetic performance of recombinant tobacco Rubisco varies depending on the SSU composition (Lin *et al.*, 2019, Preprint). However, recombinant enzymes might vary slightly in performance due to as yet unreconciled post-translational modifications that occur *in vivo*, and cannot inform the impact of organ-specific localization or developmental-specific expression of specific Rubisco isoforms (Laterre *et al.*, 2017; Lin *et al.*, 2019, Preprint). In Arabidopsis, the SSU-encoding gene family comprises four members: *rbcS1A*, *rbcS1B*, *rbcS2B*, and *rbcS3B*. Only one gene (1A) is expressed in root tips, while another (1B) is exclusively expressed in the lower side of the leaf, is unresponsive to light pulses, and lacks the light regulatory elements found in the promotors of the other three (Dedonder *et al.*, 1993; Sawchuk *et al.*, 2008). Since *rbcS1B* also shows the smallest contribution to overall SSU expression and has been replaced with a duplicate copy of *rbcS2B* in several global accessions, it is easy to discount any functional importance of this isoform (Schwarte and Tiedemann, 2011). However, the crystal structure of Arabidopsis Rubisco captured a homogenous SSU composition of only this supposed low abundant isoform (Valegård *et al.*, 2018). Khumsupan *et al*. were unable to isolate a 1B mutant in conjunction with any other SSU isoform in Arabidopsis, presenting compelling evidence that there is a key contributory role for this least expressed 1B isoform during early development.

## Measuring Rubisco in the leaf

Perhaps the most exciting aspect of the plants generated by Khumsupan *et al.* (2020) is their potential to be used to examine the impact of homogenous SSU populations on Rubisco activity *in vivo*. This bypasses not only issues of recombinant enzyme performance, but also the assumptions that *in vitro* assays make about *in vivo* conditions, such as pH, CO_2_ concentration, and molecular crowding environment at the site of carboxylation. The widely used parameterization of C_3_ photosynthesis in response to temperature has been determined using both antisense tobacco and Arabidopsis plants containing ~15% and 40% Rubisco content, respectively (Bernacchi *et al.*, 2001; Walker *et al.*, 2013). However, apart from reductions in Rubisco content, do all small subunit knockdowns have the same impact on enzyme performance? Although a full kinetic parameterization requires measurements of CO_2_ response curves over a range of oxygen partial pressures, the *in vivo* rate of Rubisco carbon assimilation (*k*cat_CO2_) may be estimated if both the maximum rate of carboxylation and the Rubisco content of the leaf are known (Box 1). This representation normalizes the carboxylation activities calculated by Khumsupan *et al.* to account for the amount of Rubisco in the leaves and suggests that, in the absence of differences in Rubisco activation, Rubisco carboxylation activity could be increased by 6–13% over the wild-type activity in *2b3b*, *1a2b*, and *1a3b* plants (Box 1). As previously shown (von Caemmerer *et al.*, 1994; Walker *et al.*, 2013), rates of Rubisco carboxylation estimated *in vivo* are greater than the *in vitro* measurements from similar T-DNA insertion lines (Atkinson *et al.*, 2017), and probably drastically overestimate the *in vivo* activity of the *1a2b3b* line containing only the 1B isoform. These differences could reflect unreconciled changes in activation state in the mutant lines, but this cannot explain the much higher estimate from line *1a2b3b.* The Rubisco turnover rate required to maintain the leaf carboxylation rate (*V*_Cmax_) observed for the *1a2b3b* line is more than triple that of any other line. Though estimates of *in vitro V*_Cmax_ (i.e. Rubisco content×*in vitro k*cat_CO2_) are much lower than observed *V*_Cmax_, suggesting that the *in vitro k*cat_CO2_ may be different for the 1B only isoform, they are unlikely to be as different as predicted here (Box 1). The discrepancy could reflect slight differences between the leaves sampled for biochemical analysis and those used for physiological measurements, and may be further compounded by the estimation method Khumsupan *et al.* (2020) used to derive *V*_Cmax_ from their *A/C*_i_ curves.

Box 1. **Differences in carboxylation rates relative to Rubisco content**

Here, the maximum rate of carboxylation (*V*_Cmax_) of lines described by Khumsupan *et al.* are plotted against Rubisco content. The dotted line represents the predicted *V*_Cmax_ at each site concentration for the estimated *in vivo k*cat_CO2_ inferred for wild-type (WT) plants. Barring differences in activation state between lines, *in vivo k*cat_CO2_ can be estimated as *V*_Cmax_/[Rubisco], while *in vitro V*_Cmax_ can be estimated as [Rubisco]×*in vitro k*cat_CO2_ (3.5 s^–1^; Boyd *et al.*, 2018). All four mutant lines targeting more than one small subunit isoform fall above this line, which could be due to increased *in vivo k*cat_CO2_.

**Table:** 

	Rubisco concentration (µM catalytic sites m^–2^)	*V*_Cmax_ (µmol CO_2_ m^–2^ s^–1^)	Estimated *in vivo k*cat_CO2_ (s^–1^)	Estimated *in vitro V*_Cmax_ (µmol CO_2_ m^–2^ s^–1^)
WT	10.0	55.5	5.5	35.0
*2b3b*	6.3	36.9	5.9	22.1
*1a2b*	5.8	36.3	6.2	20.3
*1a3b*	3.9	24.6	6.3	13.7
*1a2b3b*	0.29	5.4	18.6	1.02



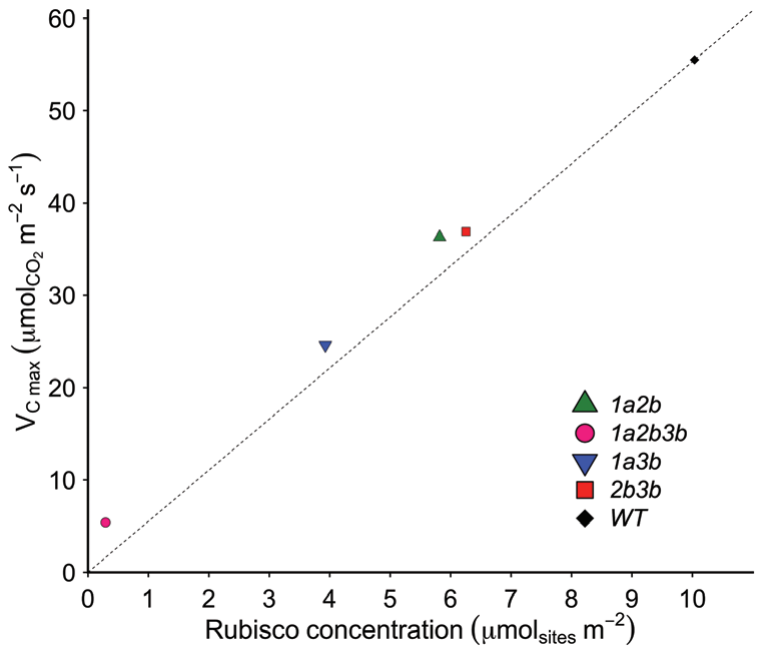

Estimated increases in the Rubisco carboxylation rate among the double mutants are small relative to the wild type and are obscured by the accompanying 37–61% reductions seen in leaf Rubisco content. However, when their impact is modelled based on similar Rubisco concentrations in the leaves, as could be achieved through simultaneous overexpression of specific Rubisco SSUs and accessory proteins (Salesse-Smith *et al.*, 2018; Yoon *et al.*, 2020), then the impact of these small differences in Rubisco activity on leaf carbon gain is apparent over a broad temperature range (Box 2). Apart from the triple mutant, this limited analysis suggests that Arabidopsis Rubiscos lacking the SSU 1A isoform may provide the most beneficial impact on modelled leaf assimilation, which is notable as this is the SSU isoform targeted via the antisense construct in plants used to derive our current *in vivo* Rubisco temperature responses in Arabidopsis (Walker *et al.*, 2013).

Box 2. **Predicting the impact of varied Rubisco activity on leaf carbon gain**

Theoretical impact of differences in Rubisco *in vivo k*cat_CO2_ on Rubisco-limited net photosynthesis if modelled at an equal leaf Rubisco concentration. Temperature responses are modelled here at 400 p.p.m. CO_2_ using the *in vivo* modelling parameters and temperature responses for Arabidopsis from Walker *et al.* (2013), and assuming an equal Rubisco site concentration, activation state, and stomatal and mesophyll conductance among all lines. The inset shows the response of the triple mutant generated and described by Khumsupan *et al.* (2020).

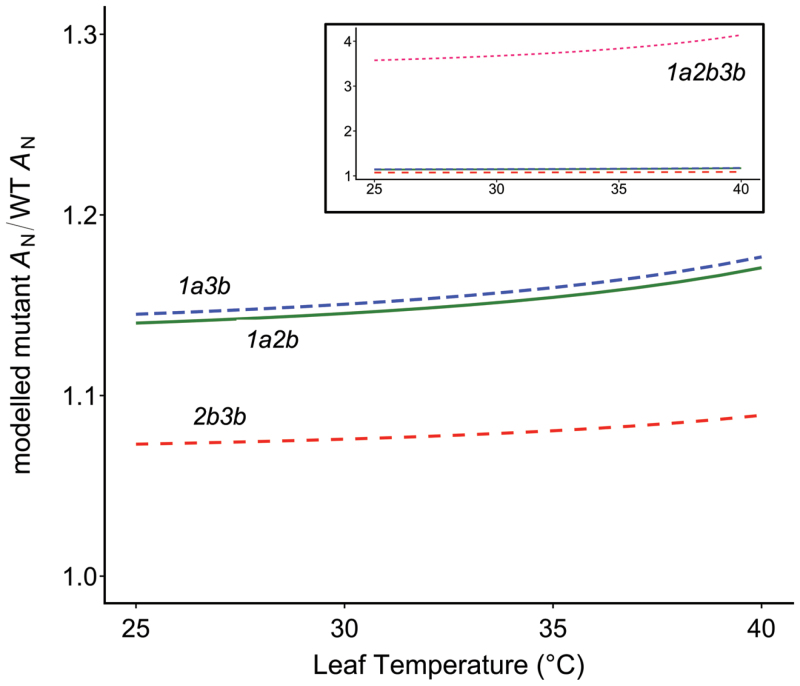

## Future perspectives

In using CRISPR/Cas9 to establish plants with a homogenous Rubisco composition, Khumsupan *et al.* (2020) have presented additional evidence that 1B has a greater impact on Rubisco than previously considered, which suggest as yet unexplored roles for Rubisco isoforms in plant development. Importantly, the generation of novel mutants described here offers a compelling new tool to address the longstanding question of the impact on Rubisco SSU composition on holoenzyme activity *in vivo*.
